# Pyrocincholic acid 3*β*-*O*-*β*-d-quinovopyranosyl-28-*O*-*β*-d-glucopyranoside suppresses adipogenesis and regulates lipid metabolism in 3T3-L1 adipocytes

**DOI:** 10.1007/s13659-017-0127-9

**Published:** 2017-05-19

**Authors:** Li Peng, Yanting Lu, Yuhui Xu, Jing Hu, Fang Wang, Yumei Zhang, Wenyong Xiong

**Affiliations:** 1 State Key Laboratory of Phytochemistry and Plant Resources in West China, Kunming Institute of Botany, Chinese Academy of Sciences, Kunming, 650201 China; 2 Key Laboratory of Tropical Plant Resources and Sustainable Use, Xishuangbanna Tropical Botanical Garden, Chinese Academy of Sciences, Kunming, 650223 China; 3 University of the Chinese Academy of Sciences, Beijing, 100049 China; 4 Yunnan Key Laboratory of Natural Medicinal Chemistry, Kunming, 650201 China

**Keywords:** Pyrocincholic acid 3*β*-*O*-*β*-d-quinovopyranosyl-28-*O*-*β*-d-glucopyranoside, Adipogenesis, Lipid metabolism, AMP-activated protein kinase

## Abstract

**Abstract:**

Obesity is crucially involved in many metabolic diseases, such as type 2 diabetes, cardiovascular disease and cancer. Regulating the number or size of adipocytes has been suggested to be a potential treatment for obesity. In this study, we investigated the effect of pyrocincholic acid 3*β*-*O*-*β*-d-quinovopyranosyl-28-*O*-*β*-d-glucopyranoside (PAQG), a 27-nor-oleanolic acid saponin extracted from *Metadina trichotoma*, on adipogenesis and lipid metabolism in 3T3-L1 adipocytes. The 3T3-L1 pre-adipocytes were incubated with vehicle or PAQG for 6 days in differentiation process. PAQG significantly reduced the adipogenesis, adiponectin secretion and the expression level of key transcription factors related to adipogenesis, such as PPARγ, C/EBPβ, C/EBPα, and FABP4. Moreover, PAQG increased the levels of FFA and glycerol in medium and reduced TG level in mature adipocytes. Interestingly, PAQG not only promoted the activation of AMPK and genes involved in fatty oxidation including PDK4 and CPT1a, but also inhibited those genes involved in fatty acid biosynthesis, such as SREBP1c, FAS, ACCα and SCD1. In conclusion, PAQG inhibits the differentiation and regulates lipid metabolism of 3T3-L1 cells via AMPK pathway, suggesting that PAQG may be a novel and promising natural product for the treatment of obesity and hyperlipidemia.

**Graphical Abstract:**

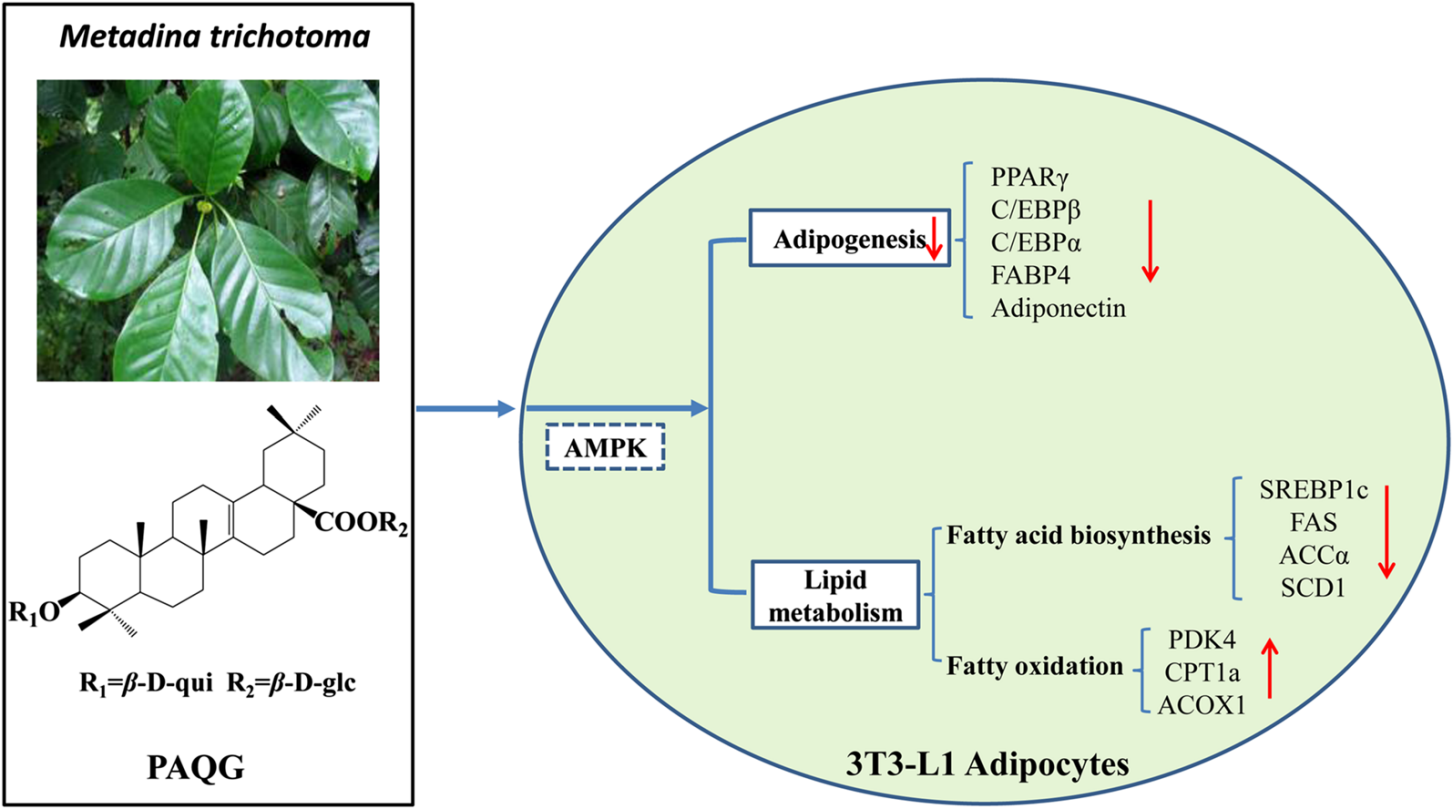

**Electronic supplementary material:**

The online version of this article (doi:10.1007/s13659-017-0127-9) contains supplementary material, which is available to authorized users.

## Introduction

Obesity has become a serious health problem around the world [1], which leads to the increasing of metabolic diseases, such as type 2 diabetes, cardiovascular disease and cancer [2]. The alterations of adipocyte in number (hyperplasia) or size (hypertrophy) are the major factors causing obesity [3]. Thus, therapeutic strategies inhibiting the adipogenesis and lipid accumulation are effective approaches for prevention and treatment of obesity and related diseases.

Many approaches are used for the treatment of obesity and related diseases, such as appetite-suppressing, inhibiting fat absorption and promoting fat oxidation. However, many anti-obesity drugs are withdrawn due to their useless effects or serious side effects [4, 5]. In recent years, several efficient and safe natural products extracted from botany, such as quercetin [6], ursolic acid [7] and a citrus bergamia extract (flavonoid compounds) [8], show a lot of advantages for the treatment of obesity.

*Metadina trichotoma* belongs to the Rubiaceae and is the unique species in the genus *Metadina*, which spreads widely in Southwest China, Vietnam, and India etc. [9]. Pyrocincholic acid 3*β*-*O*-*β*-d-quinovopyranosyl-28-*O*-*β*-d-glucopyranoside (PAQG), extracting from *Metadina trichotoma,* is a 27-nor-oleanolic acid saponin. The oleanolic acid saponin is a pentacylic triterpenoid with a oleanolic acid aglycone, which is widely distributed in food and plants and has various biology activities, such as anti-tumor, anti-virus and heptoprotective etc. [10]. Previous research demonstrated that platycodin, an oleanolic acid saponin, could decrease rat blood lipid and inhibit obesity [11], therefore we speculated that PAQG may possess the similar activity.

The 3T3-L1 murine pre-adipocytes cell line is one of the most well-known and reliable models to investigate the molecular mechanism of adipogenesis and cellular lipid metabolism [12]. Confluent 3T3-L1 pre-adipocytes can differentiate into mature adipocytes when they are exposed to the differentiation inducers such as 3-isobutyl-1-methylxanthine (IBMX), dexamethasone (DEX), insulin and rosiglitazone (Rosi). Inhibiting the differentiation process of pre-adipocytes to reduce the generation of fatty acid is an effective approach to improve obesity. In addition, promoting lipolysis is an efficient way to decrease the lipid accumulation in adipocytes, thus alleviating obesity [13].

AMP-activated protein kinase (AMPK) is a complex of three subunits (α, β and γ), which is sensitive to energy metabolism and plays a pivotal role in energy homeostasis [14]. AMPK is activated under stress and starvation, which plays a vital role in both survive and type 2 diabetes, obesity, cancer and lifespan etc. [15]. Previous studies showed that AMPK activation could inhibit the differentiation of pre-adipocytes, induce the reduction of lipid accumulation and acceleration of lipolysis [14, 16, 17], which are pivotal signaling pathway for the treatment of obesity. In this study, PAQG inhibited the differentiation of 3T3-L1 pre-adipocytes and reduced the lipid accumulation in adipocytes, it may exert the effects via promoting AMPK pathway, suggesting a novel and potent candidate in drug development.

## Results

### PAQG Suppresses the Adipogenesis in 3T3-L1 Cells

**Fig. 1:**
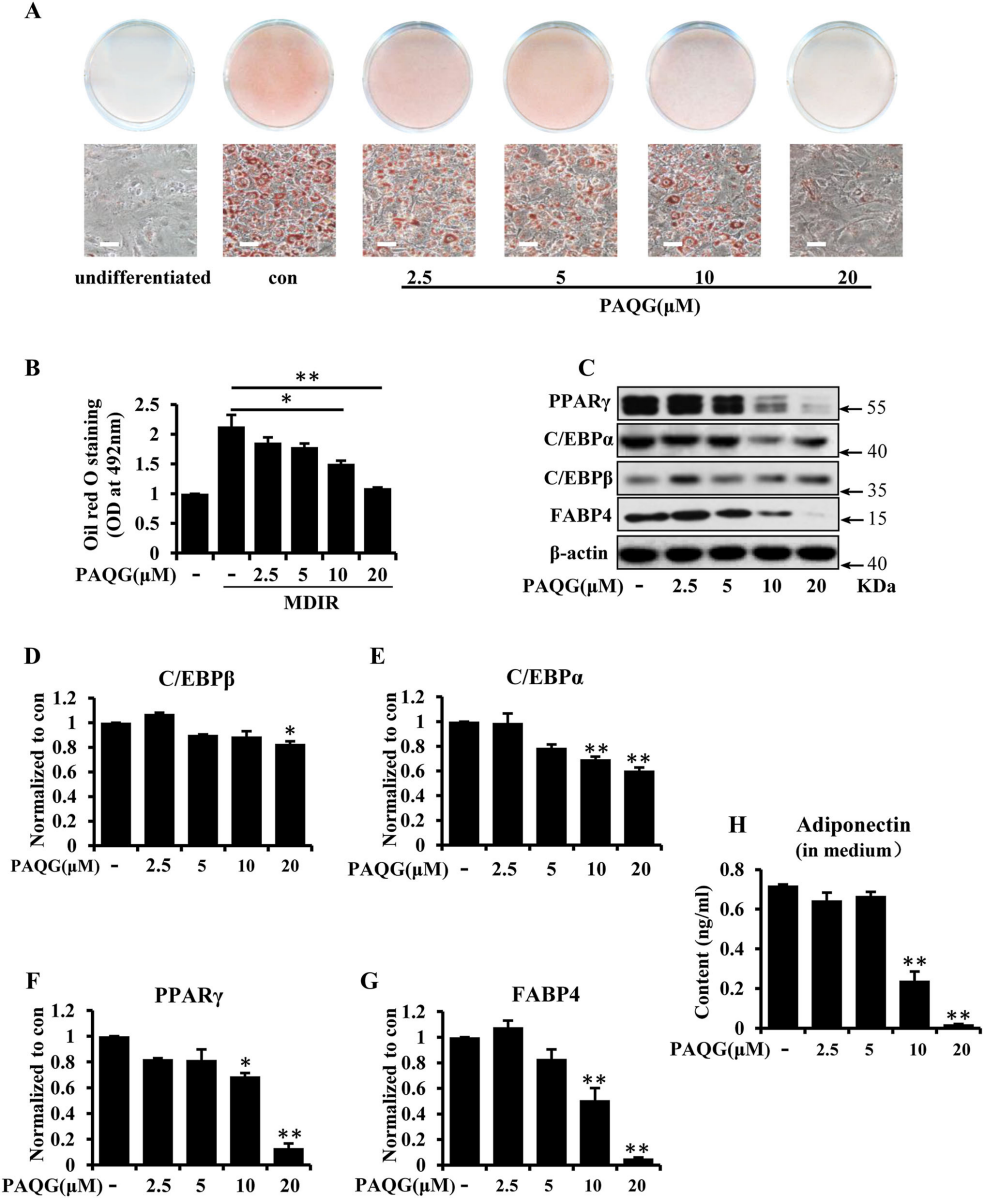
PAQG inhibites adipogenesis in 3T3-L1 cells. 3T3-L1 pre-adipocytes were incubated with indicated concentrations of PAQG or vehicle for 6 days. **a** The cells were stained with oil red O and imaged. **b** Oil Red O staining was quantitatively analyzed. **c** The expressions of PPARγ, C/EBPα, C/EBPβ and FABP4 were assessed by western blotting. **d–g** Quantification of PPARγ, C/EBPα, C/EBPβ and FABP4 levels of (**c**). **h** Adiponectin level in medium were measured by ELISA. Data are presented as mean ± SEM (*p < 0.05, **p < 0.01) from three independent experiments

The 3T3-L1 cell, as a fibroblast cell from mice, can differentiate into adipocyte in a given condition [18, 19]. Also, it has been widely used as an effective model for the identification of new anti-obesity compounds [20]. In order to test the effect of PAQG (Fig. S1A) on the differentiation of 3T3-L1 cells, we measured the level of adipocytes by Oil Red O staining. As shown in Fig. 1a, b, PAQG dose-dependently inhibited the differentiation of 3T3-L1 cells, and the inhibition effect of PAQG at 20 μM was comparable to the undifferentiated state of the cells. 20 μM PAQG did not affect the viability of 3T3-L1 and L6 cells (Fig. S1B and C).

Next, we detected the level of lipogenesis-related proteins including C/EBPβ, C/EBPα, PPARγ and FABP4 [18, 21]. As shown in Fig. 1c–g, these regulators were strikingly reduced following PAQG treatment. Furthermore, as adiponectin is a pivotal factor secreted by mature adipocytes, we measured the concentration of adiponectin in medium and the result showed that adiponectin in the medium was reduced after PAQG exposure (Fig. 1h).

### PAQG Suppresses the Early Initiation of Adipocyte Differentiation

**Fig. 2:**
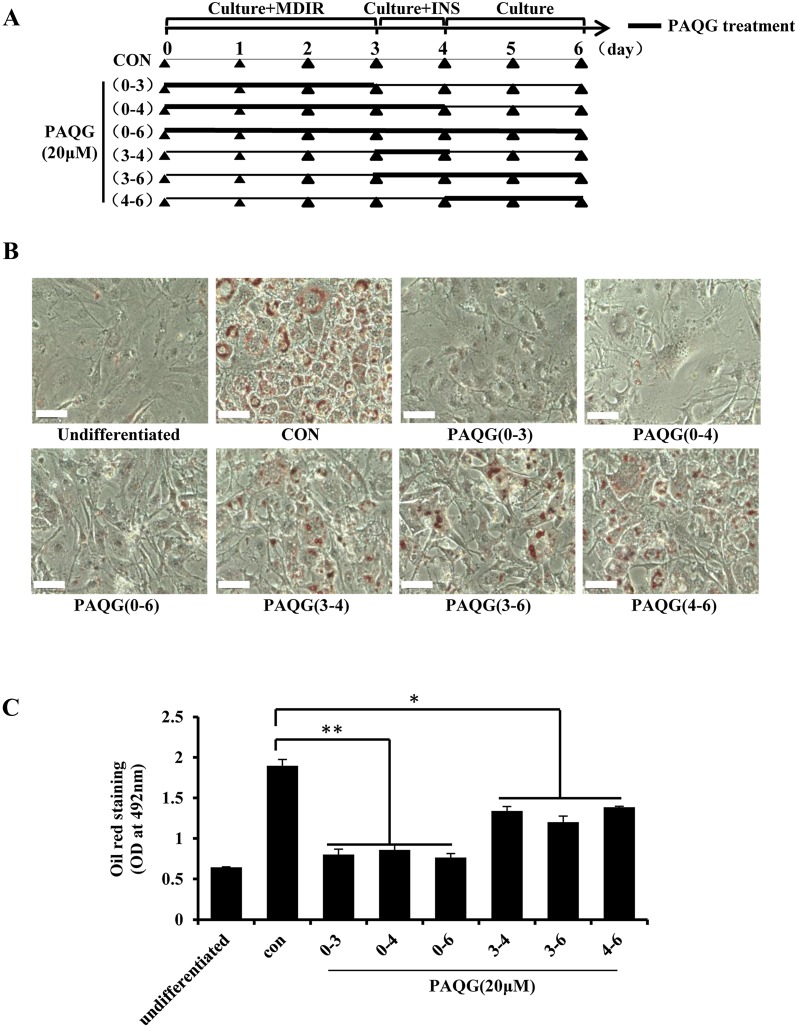
PAQG suppresses early initiation of adipogenesis. **a** 3T3-L1 pre-adipocytes were incubated with 20 μM PAQG for the indicated time periods during induction of the adipogenesis. **b** Oil Red O staining of 3T3-L1 cells samples collected at day 6 of (**a**). **c** Oil Red O staining was quantitatively analyzed. Data are presented as mean ± SEM (*p < 0.05, **p < 0.01) from three independent experiments

In order to investigate the inhibition effect of PAQG on the differentiation of pre-adipocytes, we treated the 3T3-L1 pre-adipocytes with 20 μM PAQG at different stages of adipogenesis as shown in Fig. 2a. On day 6, the differentiation level of 3T3-L1 pre-adipocytes was detected by Oil Red O staining. The accumulation of triglyceride (TG) was strikingly inhibited down to the level of un-differentiation cells by PAQG treatment on day 0–3, day 0–4 and day 0–6 (Fig. 2b, c). Interestingly, the TG accumulation was also significantly decreased when cells were exposed to PAQG on day 3–4, day 3–6 and day 4–6 (Fig. 2b, c), indicating that PAQG may regulate lipid metabolism.

### PAQG Regulates Lipid Metabolism In Adipocytes

**Fig. 3:**
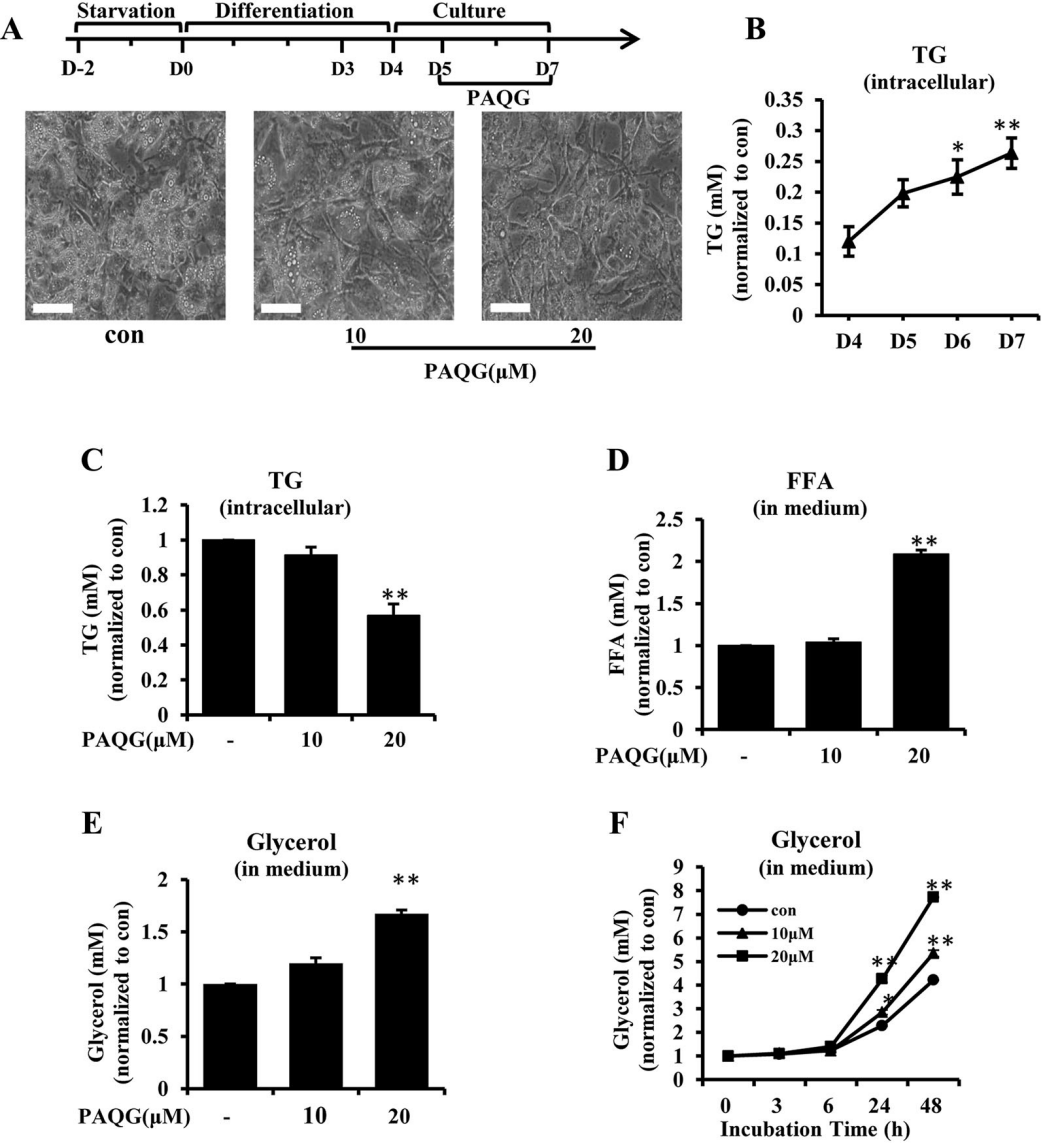
PAQG regulates lipid metabolism in adipocytes. Differentiated adipocytes were treated with indicated concentrations of PAQG for 48 h. **a** The treatment protocol (up panel) and photographs (down panel). **b**, **c** The TG levels in differentiated 3T3-L1 cells, which were treated with various concentrations of PAQG or vehicle respectively. **d**, **e** The amount of FFA and Glycerol released into medium. **f** Time-dependent effects of PAQG on glycerol release. Data are presented as mean ± SEM (*p < 0.05, **p < 0.01) from three independent experiments

To investigate the effect of PAQG on lipid metabolism in adipocytes, the differentiated adipocytes on day 5–7 were treated with PAQG at various concentrations (Fig. 3a up panel). As shown in Fig. 3a (down panel), the lipid droplet was decreased after PAQG treatment, and the cell morphology was changed to be more shuttle-like instead of roundness, which was similar to the pre-adipocytes. Next, we quantified the level of TG in differentiated 3T3-L1 cells on day 5–7 (Fig. 3b, c), the results demonstrated that TG level was significantly reduced in PAQG treated cells. As free fatty acid (FFA) and glycerol are the degradation products of TG, we detected the level of FFA and glycerol in the medium. Compared with the vehicle treatment, PAQG dose-and-time dependently induced the increase of FFA and glycerol in the medium (Fig. 3d–f).

### PAQG Activates AMPK Pathway in 3T3-L1 Adipocytes

**Fig. 4:**
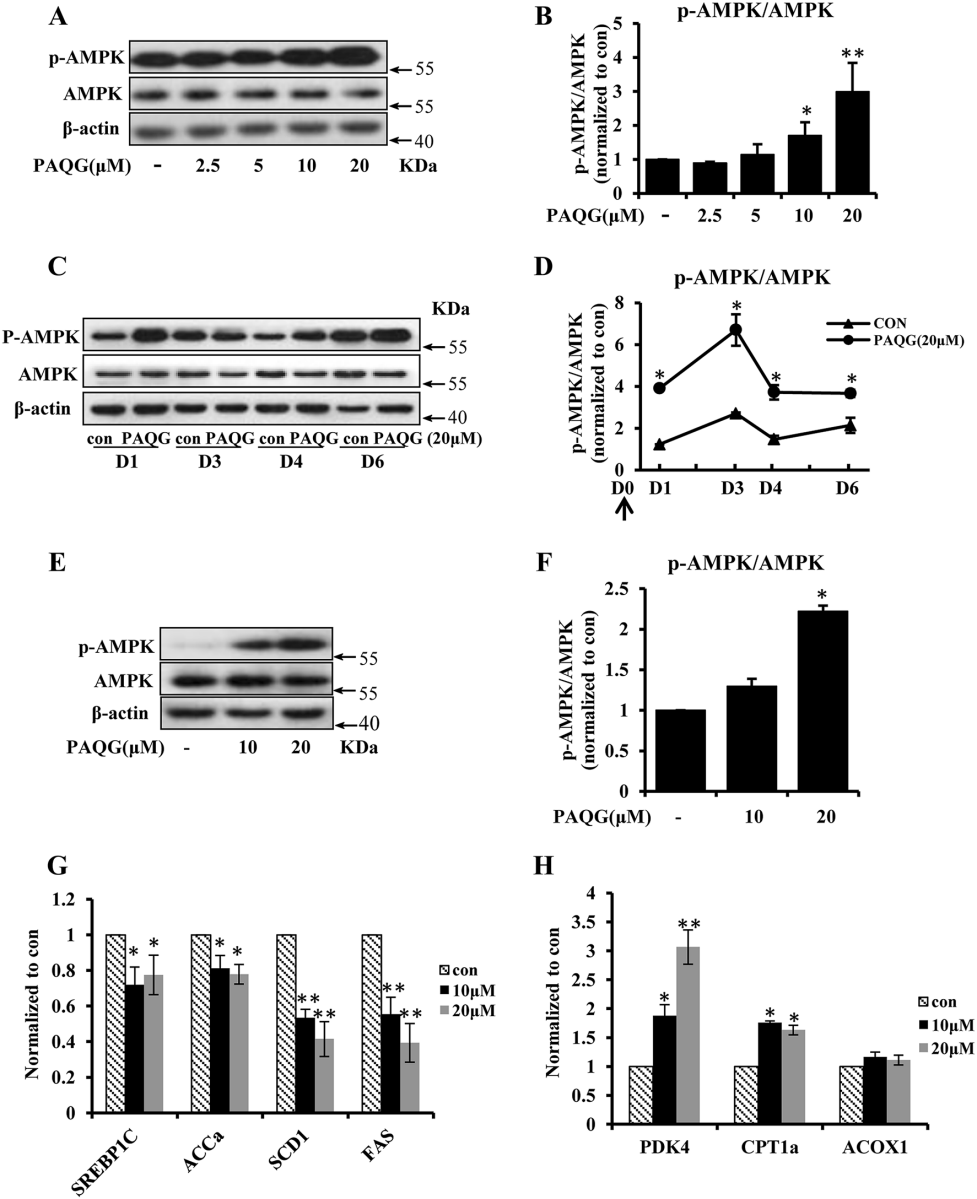
PAQG activates AMPK pathway in 3T3-L1 adipocytes. **a** 3T3-L1 pre-adipocytes were induced to differentiate in the presence of indicated concentrations of PAQG. 6 days later, the expression of AMPK and p-AMPK were assessed by western blotting and quantitatively analyzed (**b**). **c** 3T3-L1 pre-adipocytes were incubated in differentiation medium with 20 μM PAQG or vehicle, the expression of AMPK and p-AMPK were detected at different time during differentiation and quantitatively analyzed (**d**) (the arrow indicates PAQG treatment). Differentiated 3T3-L1 adipocytes were incubated with indicated concentrations of PAQG for 48 h. **e** The expression of AMPK and p-AMPK were assessed by western blotting and quantitatively analyzed (**f**). **g** The expression of AMPK target genes, (SREBP1c, FAS, ACCα, SCD1) which were connected with fatty acid biosynthesis by real-time PCR. **h** The expressions of (PDK4, CPT1a, ACOX1) by real-time PCR. Data are presented as mean ± SEM (*p < 0.05, **p < 0.01) from three independent experiments

Former study demonstrated that the phosphorylation of AMPK (p-AMPK) could inhibit the differentiation of pre-adipocytes [16, 22]. In this study, we detected the level of p-AMPK in cells treated with various concentrations of PAQG, and the results showed that p-AMPK in the differentiation process of 3T3-L1 was upregulated dose-dependently by PAQG (Fig. 4a, b). In order to determine the effect of PAQG on the level of p-AMPK in the differentiation process of 3T3-L1, we treated the cells with 20 μM PAQG or vehicle, and collected cell samples at different time of the process. The results showed that AMPK was continuously activated in the differentiation process (Fig. 4c, d).

Furthermore, differentiated 3T3-L1 adipocytes were exposed to 20 μM PAQG or vehicle for 48 h and then the cell sample were collected to detect the level of p-AMPK. Compared with vehicle treatment, PAQG increased the level of p-AMPK dose dependently (Fig. 4e, f). Moreover, p-AMPK target genes related to the fatty acid biosynthesis, including sterol regulatory element-binding transcription factor 1 (SREBP1c), fatty acid synthase (FAS), acetyl-CoA carboxylase (ACC)α and stearoyl-CoA desaturase (SCD)1 were significantly decreased by PAQG treating (Fig. 4g). In addition, the genes related to the fatty oxidation, including pyruvate dehydrogenase kinase 4 (PDK4) and carnitine palmitoyltransferase (CPT) 1a were increased at the same time (Fig. 4h).

### PAQG Promotes the Phosphorylation of AMPK

**Fig. 5:**
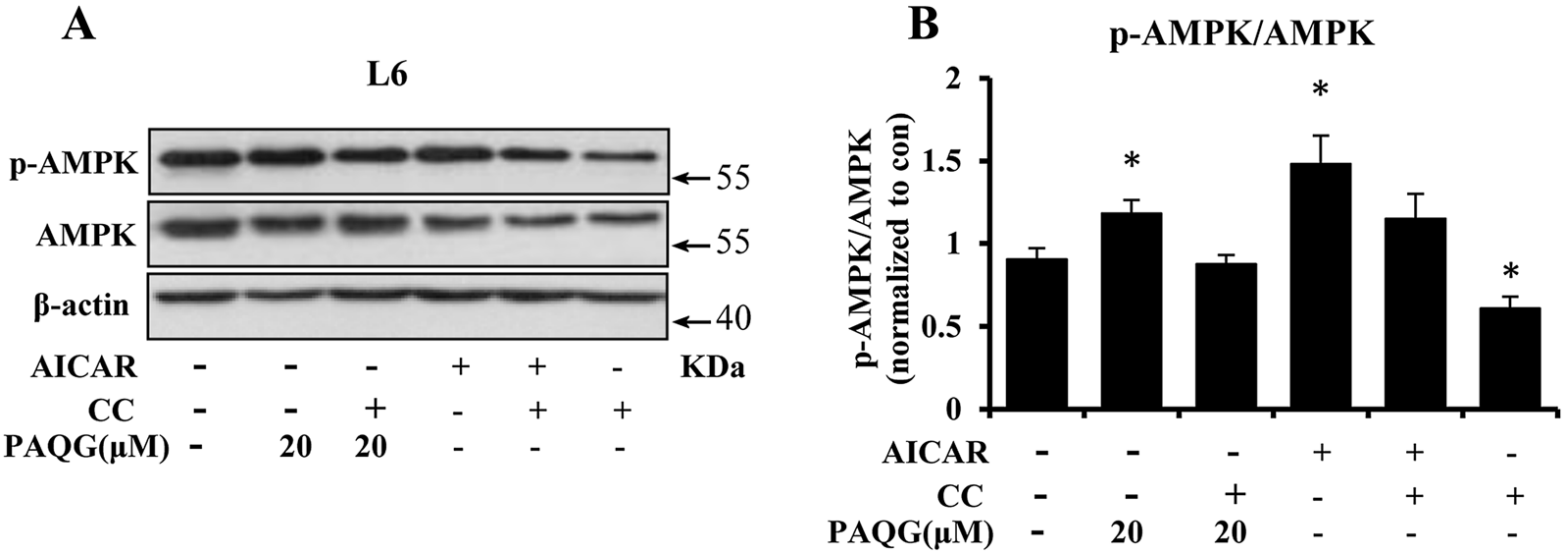
PAQG promotes phosphorylation of AMPK. **a** The effects of PAQG in the absence or presence of AICAR or compound C on the protein expression of AMPK and p-AMPK in L6 cells. **b** The results of western blotting were quantitatively analyzed. β-Actin was served as endogenous control. Data are presented as mean ± SEM (*p < 0.05) from three independent experiments

To further illustrating the effect of PAQG on the activation of AMPK, we exposed L6 cells with AICAR and compound C, specific activator and inhibitor of AMPK, respectively [16]. Compared to the vehicle treatment, compound C markedly reduced the activation of AMPK. Inversely, AICAR, PAQG, and combined use of them promoted the activation of AMPK (Fig. 5a, b). However, compound C impeded AMPK activation mediated by PAQG or AICAR, and this effect was comparable to the vehicle treatment (Fig. 5a, b).

## Discussion

Our study firstly revealed that PAQG inhibited the differentiation of 3T3-L1 pre-adipocytes (Fig. 1a–h) and the lipid accumulation in differentiated adipocytes (Fig. 3a–f) via regulating AMPK signal pathway (Fig. 4a–h).

For the inhibition of adipogenesis, PAQG down-regulated C/EBPα/β, PPARγ, FABP4 expression and adiponectin release. C/EBPβ was activated in the early stage of differentiation, and then C/EBPβ regulated the expression of downstream factors C/EBPα, FABP4 and PPARγ, finally leading to pre-adipocytes differentiated into adipocytes [23]. The protein level of adiponectin was a symbol of fat mass. In our results, PAQG also strikingly decreased lipid droplets (Fig. 3a, b), and this result further confirmed by TG test assay (Fig. 3c). In agreement with the results, the levels of FFA and glycerol in medium were increased (Fig. 3d–f). TG was hydrolyzed to FFA and glycerol, and then FFA was released to medium. The process is a well-known classic lipolysis path.

SREPB1c is a key transcriptional factor in downstream of AMPK pathway in lipid metabolism, which facilitates lipid synthesis together with ACCα, SCD1 and FAS [24]. CPT1a transports long chain fatty acids into mitochondria for beta oxidation, thus acts as a key regulatory enzyme in modulating fatty acid oxidation [7]. In current study, we found that AMPK, an important regulatory factor in lipolysis process [17], is phosphorylated and activated (Fig. 4e, f) by PAQG. The down-regulation of SREBP1c, FAS, SCD1, ACCα and the up-regulation of CPT1a and PDK4 support the effect of PAQG on AMPK pathway in inhibiting lipid synthesis and promoting oxidation. In addition, for the purpose of confirming the effect of PAQG on AMPK activation, we applied AICAR as a positive control and compound C as a negative control to explore the effect of PAQG on AMPK activation. As shown in Fig. 5, these results suggest that PAQG can activate AMPK. Although we verified that PAQG inhibited differentiation and lipid accumulation by AMPK signal pathway, the direct target protein of PAQG in AMPK pathway is still unknown.

In conclusion, our results demonstrated that PAQG inhibited 3T3-L1 cells differentiation and lipid accumulation in mature adipocytes by regulating the expression of transcriptional factors, related genes of fatty acid synthesis and fat oxidation via regulating activity of AMPK pathway. And, PAQG might be a promising anti-obesity natural product for treating type 2 diabetes and other metabolic diseases.

## Materials and Methods

### Chemical Reagents

Dulbecco’s modified Eagle’s medium (DMEM)/high glucose was obtained from Biological Industries (Israel). Calf serum (CS) for cell culture was purchased from GIBCO BRL (Grand Island, NY, USA). Penicillin/streptomycin (P/S) and fetal bovine serum (FBS) were obtained from Biological Industries (Israel). Rosiglitazone (Rosi), dimethyl sulfoxide (DMSO) and 3-isobutyl-1-methylxanthine (IBMX) were obtained from Sigma-Aldrich (St Louis, MO, USA). Insulin was from Roche (Switzerland), and dexamethasone (DEX) was from Adamas (Switzerland). The 5-amino-4-imidazolecarboxamide ribonucleotide (AICAR) and cell lysis buffer were from Beyotime (Shanghai, China). Compound C was purchased from Calbiochem (San Diego, CA). Bovine serum albumin (BSA) was from Shanghai Sangon Biotech Co., Ltd. (Shanghai, China). The antibodies against AMPK, phospho-AMPK-α (Thr172), FABP4, C/EBPβ, C/EBPα and PPARγ were from Cell Signaling Technology (Beverly, MA, USA), β-actin was from Santa Cruz Biotechnology (Santa Cruz, CA, USA). Triglycerides Kit, Glycerol Assay Kit and Non-esterified fatty acids Kit were purchased from Nanjing Jiancheng Bioengineering Institute. Mouse Adiponectin ELISA Kit was purchased from MULTI SCIENCES.

### Cell Culture and Adipocyte Differentiation

The 3T3-L1 murine pre-adipocytes cell line was purchased from the American Type Culture Collection (ATCC, Manassas, VA, USA). Cells were routinely cultured in Dulbecco’s modified Eagle’s medium (DMEM) supplemented with 10% CS and 1% P/S at 37 °C in a 5% CO_2_ atmosphere [25]. For adipocytes differentiation, 2-day postconfluent cells (day 0) were placed in Dulbecco’s modified Eagle’s medium (DMEM) supplemented with 10% FBS, 1% P/S, IBMX, DEX, insulin [26] and Rosi (designated hereafter as MDIR). After 3 days (day 3), the medium was changed to post-differentiation medium (10% FBS-DMEM containing 1 μg/ml insulin) for one day (day 4), and then replaced with 10% FBS-DMEM for 2 days. 3T3-L1 cells were treated with PAQG in two different methods for different purposes. The first one: PAQG was added at day 0 to day 6 during the differentiation process as depicted above. The second one: PAQG was added at day 5 and cells were harvested after 2 days treatment. The PAQG was dissolved in DMSO, 3T3-L1 cells cultured in DMEM supplemented with 0.1% DMSO were used as a vehicle control for all experiments [25].

### Cell Viability Assay

Cell viability was measured by the Cell Proliferation MTS Kit (Promega Corporation, Madison) as introduced by the manufacturer. In brief, the 3T3-L1 mature adipocytes were treated with various concentrations of PAQG in 96-well plates for 6 days. The medium was removed, and the 20% of MTS solution was added to each well for 4 h. The absorbance was measured at 492 nm by using a microplate reader (Perkin Elmer Envision Multilabel reader). The method used to detect L6 cell viability is similar.

### Oil Red O Staining

Oil Red O is a lysochrome diazo dye (fat-soluble) used for staining of neutral triglycerides and lipids [8]. The Oil Red O working solution was prepared as described [25, 27]. At a word, 3T3-L1 cells were washed twice with phosphate-buffered saline (PBS) after removed the culture medium and were fixed in 10% formaldehyde at room temperature for 10 min, the 10% formaldehyde was discarded and new 10% formaldehyde was added for 1 h. Wash twice with water and one time with 60% isopropanol sequentially after fixing. The 3T3-L1 cells were stained with Oil Red O working solution for 30 min at room temperature. The stained cells were washed with water five times to wash the non-specific binding dye away and then taken photos under microscopy. In order to quantify the quantity of lipid droplets, the stained 3T3-L1 cells were washed with 100% isopropanol and the specific binding Oil Red O dissolved in it, the OD was measured at 492 nm by a microplate reader (Perkin Elmer Envision Multilabel reader).

### Measurement of Triglycerides, Free Fatty Acids and Glycerol

After treatment with PAQG for 48 h, the medium was collected to detect free fatty acids (Non-esterified fatty acids Kit, Biosino Bio-Technology and Science Incorporation, China) and glycerol (Glycerol Assay Kit, Nanjing Jiancheng Bioengineering Institute, China), and the intracellular lipid was collected by isopropanol to detect triglycerides (Triglycerides Kit, Biosino Bio-Technology and Science Incorporation, China).

### Preparation of Proteins and Western Blot Analysis

Cells were washed twice with ice-cold PBS and subsequently lysed in equal volumes of cell lysis buffer containing protein inhibitor cocktail (Sigma) for 30 min. Protein concentration was measured by BCA protein assay kit (Pierce). The lysates mixed with a certain amount of 5× loading buffer and boiled at 100 °C for 10 min. The protein was separated by 10% SDS-PAGE and transferred to PVDF membrane, blocked in 5% non-fat dried milk dissolved in PBS-T for 1 h and immunoblotted with antibodies specific for FABP4, C/EBPβ, PPARγ, C/EBPα, AMPK, phospho-AMPK-α (Thr172) and β-actin. Finally, the immunoblots were quantified using the Metamorph software and expressed as a ratio of phosphorylated to total protein or total protein to β-actin.

### RNA Isolation and Real-Time PCR for Gene Expression

RNA was extracted from 3T3-L1 cells using TRIzol reagent (TIANGEN BIOTECH), and cDNA was synthesized using cDNA synthesis kits (TIANGEN BIOTECH).

Real-time PCR cDNA gene expression was detected using the SYBR Green Master kit and a spectrofluorometric thermal cycler (Applied Biosystems). β-Actin was used as the RNA loading control. Specific primers were designed as shown in Table 1.

**Table 1:** Forward and reverse primers used for qPCR

Gene	Forward	Reverse
SREBP1c	CAGCTCAGAGCCGTGGTGA	TGTGTGCACTTCGTAGGGT
FAS	AGCTTCGGCTGCTGTTGGAAGT	TCGGATGCCTCTGAACCACTCACA
ACCα	TGAGAAGGTTCTTATCGCCAACA	TTCATAAGACCACCGACGGA
SCD1	ATGGATATCGCCCCTACGAC	GATGTGCCAGCGGTACTCAC
PDK4	AGCCCTGTCAGAGTTTGTAGAC	TGCCTTGAGCCATTGTAGGG
CPT1a	GGACTCCGCTCGCTCATT	GAGATCGATGCCATCAGGGG
ACOX1	CATGTGGTTTAAAAACTCTGTGC	GGCATGAAGAAACGCTCCTG
β-Actin	TGGAATCCTGTGGCATCCATGAAA	TAAAACGCAGCTCAGTAACAGTCC

### Statistical Analysis

All data were presented as mean ± SD of at least three separate experiments. The statistical analysis was performed by using one-way ANOVA and Dunnett’s Post hoc test. P values less than 0.05 were considered to be statistically significant.

## Electronic supplementary material

Below is the link to the electronic supplementary material.

Supplementary material 1 (DOCX 176 kb)
