# Supplementary material for: Pyrocincholic acid 3β-O-β-d-quinovopyranosyl-28-O-β-d-glucopyranoside suppresses adipogenesis and regulates lipid metabolism in 3T3-L1 adipocytes
Source: Nat Prod Bioprospect. 2017 May 19;7(3):225–34. doi: 10.1007/s13659-017-0127-9 (PMC5481272; doi:10.1007/s13659-017-0127-9)
Supplement: Supplementary file 1 — Supplementary material 1 (DOCX 176 kb) [file 13659_2017_127_MOESM1_ESM.docx]

**Supplementary**

PAQG did not affect the viability of 3T3-L1 and L6 cells.

The structure of PAQG was shown in Fig S1A. To evaluate the toxicity of PAQG on 3T3-L1 cells, the cells were treated with various concentration of PAQG for 6 days. Considering that 20 μM PAQG can distinctly inhibit 3T3-L1 pre-adipocytes differentiation (Fig 1A), 20 μM PAQG was used as a maximum dosage in our study and the maximum concentration of PAQG was 50 μM in the toxicity experiment. The cell viability was not impaired compared with control (Fig S1B), the similar method was used to evaluate the toxicity of PAQG on L6 cells (Fig S1C).


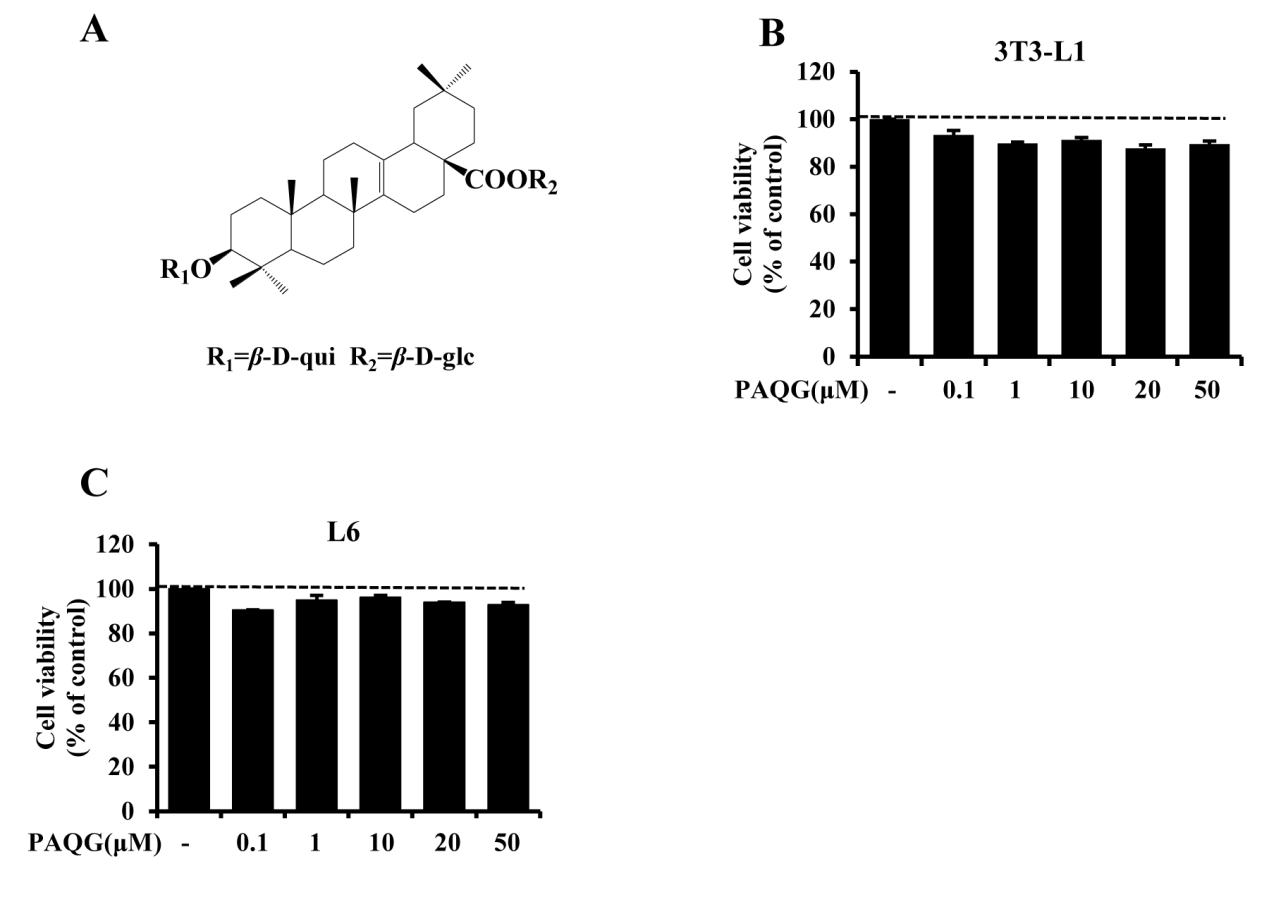


**Fig S1**. **PAQG did not affect the viability of 3T3-L1 and L6 cells.** (A) Chemical structure of compound PAQG. (B) and (C) Effect of compound PAQG on cell viability in 3T3-L1 and L6 cells, respectively.
